# Heart transplantation using a donor heart with repaired tetralogy of Fallot: a case report

**DOI:** 10.1093/ehjcr/ytad557

**Published:** 2023-11-15

**Authors:** Takura Taguchi, Daisuke Yoshioka, Koichi Toda, Shigeru Miyagawa

**Affiliations:** Department of Cardiovascular Surgery, Osaka University Graduate School of Medicine, 2-2-E1, Yamadaoka, Suita, Osaka 565-0871, Japan; Department of Cardiovascular Surgery, Osaka University Graduate School of Medicine, 2-2-E1, Yamadaoka, Suita, Osaka 565-0871, Japan; Department of Cardiovascular Surgery, Osaka University Graduate School of Medicine, 2-2-E1, Yamadaoka, Suita, Osaka 565-0871, Japan; Department of Cardiovascular Surgery, Osaka University Graduate School of Medicine, 2-2-E1, Yamadaoka, Suita, Osaka 565-0871, Japan

**Keywords:** Heart transplantation, Tetralogy of Fallot, Marginal donor, Case report

## Abstract

**Background:**

Heart transplantation is the gold standard therapy for end-stage heart failure; however, it is limited by a shortage of available donors. In recent years, heart transplantations have been performed using marginal donor hearts with valvular and/or congenital cardiac abnormalities.

**Case summary:**

A 60-year-old woman with acromegalic cardiomyopathy underwent left ventricular assist device implantation and aortic valve (AV) closure 4 years prior. After 2 months, repeat AV closure and omental flap transposition were performed. During the outpatient follow-up, the patient developed recurrent severe AV regurgitation and bacteraemia-induced subarachnoid haemorrhage. She underwent urgent heart transplantation using a marginal donor heart with preserved cardiac function, mild pulmonary valve stenosis, and regurgitation after pulmonary valve-sparing tetralogy of Fallot (TOF) repair. An anatomical anastomosis was possible. She had no signs of infection, heart failure, arrhythmia, or immune rejection 15 months after the heart transplantation.

**Discussion:**

In this case, the donor heart with repaired TOF did not require pulmonary valve replacement and was anatomically intact. Donor hearts with repaired TOF that are expected to have long-term durability in terms of cardiac function may be used for successful heart transplantations. The repair of marginal donor hearts creates an opportunity to increase the number of viable donors.

**Learning points**

- Heart transplantation is effective for left ventricular assist device patients with infection or heart failure; however, there is a shortage of donors worldwide.
- A heart with repaired tetralogy of Fallot and preserved cardiac function can be used as a donor heart for heart transplantation without anatomical complications.

## Introduction

Heart transplantation is the gold standard therapy for end-stage heart failure; however, it is limited by a shortage of available donors,^1^ leading to longer waiting times of over 1500 days in our country, even after implantation of a left ventricular assist device (LVAD).^2^ During the waiting period, patients with implantable LVAD often develop various complications such as infection, stroke, and heart failure, which are difficult to treat and often fatal.^3^ Therefore, in recent years, heart transplantations have been performed using marginal donor hearts with valvular and/or congenital cardiac abnormalities.^4^

## Summary figure

**Figure:**
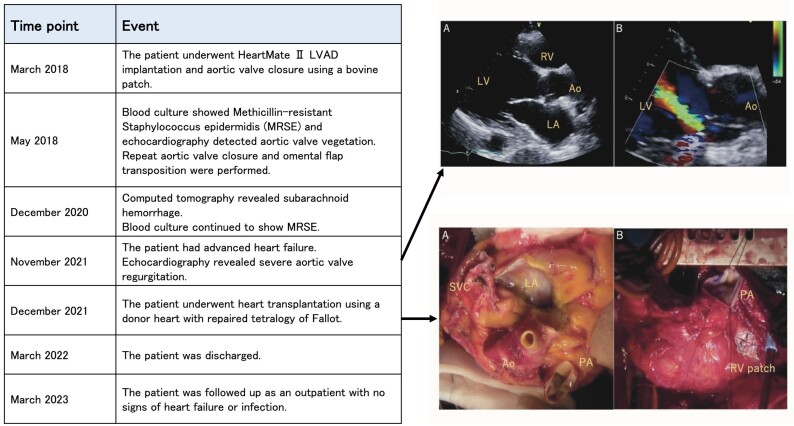


We herein report the case of a patient with LVAD implantation who experienced post-operative persistent bacteraemia and AV regurgitation (AR) and was successfully treated with heart transplantation using repaired tetralogy of Fallot (TOF) allograft.

### Case presentation

**Figure 1:**
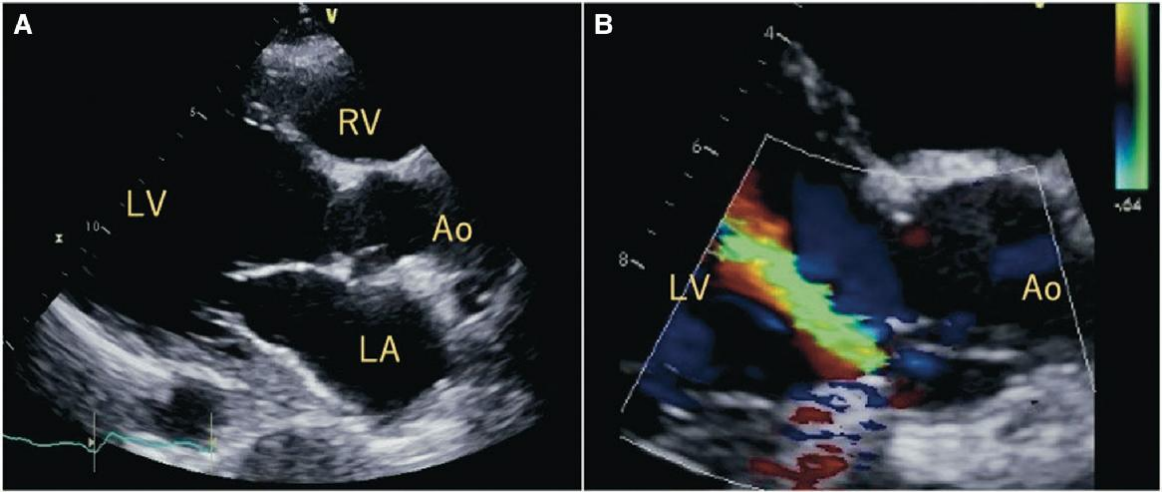
Pre-operative transthoracic echocardiography. (*A*) The left ventricle is dilated. There is no vegetation. (*B*) Severe aortic valve regurgitation is detected. Ao, aorta; RV, right ventricle; LA, left atrium; LV, left ventricle.

A 60-year-old woman with acromegalic cardiomyopathy underwent HeartMate II (Abbott Laboratories, Chicago, IL, USA) LVAD implantation and aortic valve (AV) closure using a bovine pericardial patch 4 years prior. Post-operative bacteraemia, *de novo* AR, and vegetations were observed; hence, consecutive repeat AV closure and omental flap transposition were performed. During the outpatient follow-up, the patient had recurrent severe AR (*Figure 1*) and three cerebral haemorrhages caused by bacteraemia. The patient did not develop sepsis with oral antibiotic therapy, but the activities of daily living (ADLs) were severely limited.

Laboratory test results showed the following: white blood cell count, 5260/μL; C-reactive protein levels, 2.1 mg/dL; creatine levels, 1.62 mg/dL; and brain natriuretic peptide levels, 1138 pg/mL. Blood culture results continued to show methicillin-resistant *Staphylococcus epidermidis* (MRSE). Transthoracic echocardiography (TTE) showed a low left ventricular ejection fraction (LVEF, 12%), a dilated left ventricle [left ventricular end-diastolic dimension/end-systolic dimension (LVEDD/SD), 73/69 mm], and severe peri-annular AR.

The donor, whose cause of brain death was subarachnoid haemorrhage, was a 41-year-old man, who underwent pulmonary valve-sparing TOF repair at the age of 3 years. The donor’s TOF repair preserved the pulmonary valve and did not require plication, only enlargement with pulmonary artery (PA) and right ventricle (RV) patching. After that surgery, there was no finding of heart failure and no need for reoperation. Transthoracic echocardiography revealed normal cardiac function (LVEF, 66%), a normal-sized left ventricle (LVEDD/SD, 48/31 mm), mild pulmonary valve stenosis (PS) (peak velocity 2.5 m/s), mild pulmonary valve regurgitation (PR), and no other notable findings.

**Figure 2:**
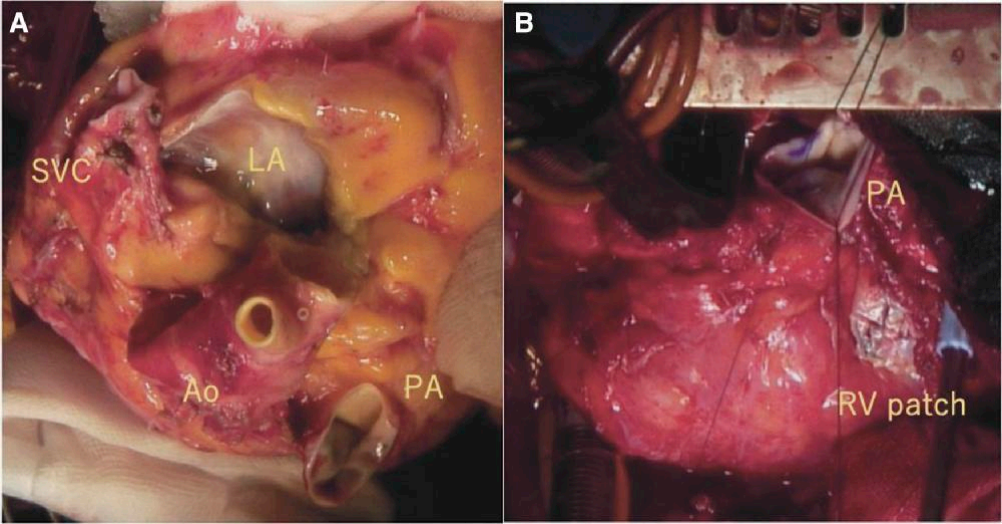
Intraoperative image of the donor heart. (*A*) The pulmonary valve is autologous, and the bicuspid valve and the Ao are anteriorly deviated. (*B*) The donor’s PA is enlarged, and there is a 2 cm square patch in front of the RV. Ao, aorta; PA, pulmonary artery; RV, right ventricle; LA, left atrium; SVC, superior vena cava.

The patient underwent heart transplantation using the modified bicaval technique. Recipient heart was resected just above the pulmonary valve for keeping the main pulmonary trunk. When the donor heart was inspected, a 2 cm square patch was placed in front of the slightly stenotic donor’s RV outflow tract, and the pulmonary valve was bicuspid valve. The ascending aorta was anteriorly deviated, but an anatomical anastomosis was possible (*Figure 2*). Weaning from the cardiopulmonary bypass was uneventful, and intraoperative right ventricular function was preserved. The donor ischaemia time was 155 min. Vegetation was found on the inside of LVAD but not at the AV patch. Bacteriological examination revealed MRSE in the vegetation.

Blood culture results on post-operative Day 2 were negative. Post-operative pancreatitis developed but improved with medical therapy. The patient was weaned off inotropic agents on post-operative Day 12. Post-operative echocardiography showed normal cardiac function (LVEF, 77%), a normal-sized left ventricle (LVEDD/SD: 42/23 mm), mild PS (peak velocity 2.8 m/s), and mild PR. Post-operative right heart catheterization (RHC) at 1 month revealed the following pressure values: right atrium (RA) 4 mmHg, RV 46/7 mmHg, PA 24/12 mmHg, cardiac output (CO) 4.5 L/min, and pulmonary vascular resistance (PVR) 1.3 Wood unit (*Table 1*). The patient was discharged on post-operative Day 116. Right heart catheterization at the 1 year follow-up after heart transplantation revealed the following: RA 5 mmHg, RV 47/6 mmHg, PA 28/12 mmHg, and CO 5.3 L/min (*Table 1*). She had no signs of infection, heart failure, arrhythmia, and immune rejection 15 months after the heart transplantation.

**Table 1:** Post-operative echocardiography and right heart catheterization

Echocardiography							
Post-transplantation	Function	Size measurements			Valve		
1 month	LVEF 77%	LVEDD/SD 42/23 mm			Mild TR Mild PR Mild PS (peak velocity 2.8 m/s)		

Right heart catheterization							
Post-transplantation	RA (mmHg)	RV (mmHg)	PA (mmHg)	PCWP (mmHg)	CO (L/min)	CI (L/min/m^2^)	PVR (Wood unit)
1 month	4	46/7	24/12	11	4.5	2.5	1.3
1 year	5	47/6	28/12	11	5.3	3.1	1.5

LVEF, left ventricular ejection fraction; LVEDD/SD, left ventricular end-diastolic dimension/end-systolic dimension; TR, tricuspid valve regurgitation; PR, pulmonary valve regurgitation; PS, pulmonary valve stenosis; RA, right atrium; RV, right ventricle; PA, pulmonary artery; PCWP, pulmonary capillary wedge pressure; CO, cardiac output; CI, cardiac index; PVR, pulmonary vascular resistance.

## Discussion

Patients with LVAD have been reported to have good long-term outcomes.^3^ However, patients who develop infection or heart failure while awaiting transplantation have a higher mortality rate and sometimes require surgical intervention. Explanting LVAD is the only method that decreases the mortality of LVAD patients with bacteraemia,^5^ who may require urgent heart transplantation. In many of these cases, the shortage of donors makes it necessary to use marginal donors. As in this case, repeated bacteraemia, subarachnoid haemorrhage, and heart failure due to severe AR after LVAD implantation required surgical intervention, but AV closure was considered high-risk. As a marginal donor heart after TOF repair with preserved cardiac function and mild PS and PR was available, we decided that urgent heart transplantation using this marginal donor heart would be the most effective treatment to improve the patient’s situation.

There have been no reported cases of heart transplantation using donor hearts with repaired TOF, and hence, pre-operative evaluation for anterior displacement of the aorta is required, and right ventricular outflow tract reconstruction must be performed, if necessary. Pulmonary valve replacement is also recommended for severe PR and/or moderate right ventricular outflow tract obstruction (RVOTO).^6^ In recipients with high PVR, simultaneous pulmonary valve replacement should be considered because right heart failure may become apparent after transplantation if the donor’s heart has PR or RVOTO. In this case, the donor heart was expected to have long-term durability without surgery and could be transplanted without anatomical difficulty. The post-operative cardiac examination showed no abnormal findings. Therefore, heart transplantation using the donor’s heart was considered appropriate, and post-operative follow-up was required, due to the repaired TOF.

The routine use of marginal donors may be considered in the future, but cardiac function after TOF repair differs from case to case. Patients with a palliative shunt before initial correction, i.e. those with Waterston shunt, Blalock–Taussig shunt, or Potts anastomosis, are more at risk of dying or needing pulmonary valve replacement than patients without them. Additionally, mortality is higher in patients who experience post-operative arrhythmias.^7^ These factors should be considered when using a heart with repaired TOF as a marginal donor.

In conclusion, this report indicates that donor hearts with repaired TOF that are expected to have long-term durability in terms of cardiac function may be used for successful heart transplantation.

## Data Availability

The data underlying this article will be shared on reasonable request to the corresponding author.

## Lead author biography

Takura Taguchi graduated from Osaka University, Japan, and acquired an MD degree in 2015. He has worked at the Department of Cardiovascular Surgery, Osaka University Graduate School of Medicine, Japan, from 2021. His principal field of interest is cardiovascular surgery, extracorporeal membrane oxygenation, ventricular assist devices, and heart transplant.
